# Editorial: Plants for future climate: responses and adaptations to combined, multifactorial, and sequential stresses

**DOI:** 10.3389/fpls.2023.1290649

**Published:** 2023-10-13

**Authors:** Vinay Kumar, Ashish Kumar Srivastava, Oksana Sytar, Suprasanna Penna

**Affiliations:** ^1^ Department of Biotechnology, Modern College of Arts, Science and Commerce, Savitribai Phule Pune University, Pune, India; ^2^ Nuclear Agriculture and Biotechnology Division, Bhabha Atomic Research Centre (BARC), Mumbai, India; ^3^ Department of Plant Biology, Institute of Biology and Medicine, Taras Shevchenko National University of Kyiv, Kyiv, Ukraine; ^4^ Institute of Plant and Environmental Sciences, Slovak University of Agriculture, Nitra, Slovakia; ^5^ Amity Centre for Nuclear Biotechnology, Amity Institute of Biotechnology, Amity University of Maharashtra, Mumbai, India

**Keywords:** combined stress, sequential stress, plant-environment interaction, transgenic technology, climate change

Climate change has a substantial bearing on crop productivity and food security, and hence there is a need to develop resilience to mitigate climate change induced impacts in crop plants (Acevedo et al., 2020; FAO, 2020; Raj et al., 2022). Under the field environment settings, crop plants are exposed to harsh fluctuating and changing climates with growing incidences of multiple abiotic stresses, that may include dual, multifactorial and/or sequential stresses. Though the responses of plants to combinations of two or more (dual or multifactorial) stresses can not necessarily be extrapolated from their responses to individual stresses. Still, historically most of the studies have been aimed at understanding and deciphering the plant responses and adaptation strategies against individual stresses ranging from deficiency/excess of water, hyper-salinity, extreme or fluctuating temperatures, harmful UV-rays and heavy metals. On the other hand, a complex interplay of multiple stresses occurs under actual field conditions. Besides, combined or sequential occurrences of abiotic stresses may have additive impacts on crop plants than their individual occurrences (Sánchez-Bermúdez et al., 2022). Yet, the complex stress responses triggered under combined or sequential stresses are largely overlooked. In recent times, there has been a renewed interest in comprehensive investigations to be undertaken aimed at understanding plant responses and adaptation strategies against combined stresses (Suprasanna, 2020; Shabbir et al., 2022), at physiological, cellular, and/or molecular levels, and how plants fine-tune their responses through transcriptional/post-transcriptional regulations and intricate regulatory networks (Govind et al., 2022). This ‘Research Topic’ is aimed at highlighting the latest advances in our understanding of plant responses and adaptation/tolerance to dual, multifactorial or sequential abiotic stresses, and also to introduce the molecular toolbox used in studying the plant responses to combined and/or sequential stresses.

Genetic dissection of quantitatively inherited traits is of paramount importance to improve the selection efficiency in crop plants. Somegowda et al. demonstrated that sorghum breeding can be directed to achieve genetic gains for both fodder biomass and digestibility without any trade-offs, suggesting the possibility for the improvement of these traits. The authors found that digestibility and grain yield did not show a significant correlation under different water regimes. The authors genotyped RIL population using the genotyping-by-sequencing technique and identified 1,141 informative single nucleotide polymorphism (SNP) markers. A linkage map was constructed, and a total of 294 QTLs were detected, with 534 epistatic interactions, across all of the traits under study. The vast majority of these were small-effect QTLs, and what breeders need are a smaller, manageable number of large effect QTLs. Since extensive mapping may hold key in crop improvement programs by undermining yield and quality. Though opportunities for marker-assisted breeding for fodder quality and nutrition exist, these are not yet mapped and this study is one of the first attempts to do so.

Combined stresses share distinctiveness and complexity of the physiological and molecular network modules including that of proline hub (Maria et al., 2021). Kishor et al. have presented a perspective review emphasizing the role of proline in high-temperature and other stress tolerance, mainly through regulating the redox potentials and, also discussed current updates, challenges and future research trends. This review provides evidence that proline is a key osmolyte, and that the proline cycle acts as a shuttle to transport redox couples from mitochondria to cytoplasm and back. Authors suggest that extensive studies are needed to unravel the exact roles, for example of proline cycle and ROS and their homeostasis in developing heat tolerance in crop plants.

The continuing threat of climate warming has led to higher winter temperatures which have a strong impact on the seed dormancy and seed germination. Seed germination and seedling development extreme vulnerability to climate change and hence, data on the cold stratification temperatures will contribute to predicting the probable responses of plant populations to future global climate change. Breaking the seed dormancy could be achieved by a sequential temperature treatment of warm and cold or cold and warm temperature treatments (Liao et al., 2021). Cheng et al. demonstrated that germination traits of seeds of *Spartina alterniflora* seeds from different provenances in China at different latitudes differed significantly in response to stratification and ambient temperature. This study evaluated the effects of cold stratification (5°C) and higher temperatures on seed germination and, found that longer cold stratification significantly promoted germination rate and germination index, but decreased germination time. These findings confirmed the stratification and temperature as the most important factors regulating the dormancy and germination seeds, attributable to drive this variation along latitude. This study also suggests that global warming may accelerate the expansion and spread of invasive species.

Salinity stress is one of the major factors leading to losses in crop productivity. Plant–bacterial interactions are known for complementing plant growth and productivity especially, under the stress conditions (Meena et al., 2023). Meza et al. isolated two plant growth-promoting bacteria (PGPB)- *Bacillus proteolyticus* Cyn1 and *Bacillus safensis* Cyn2, from the rhizospheric soil of the Chilean ecotype “Sapito” of *Phaseolus vulgaris*. Cyn1 produced NH_3_ and HCN along with secreting siderophores, while Cyn2 produced NH_3_ and siderophores. Both the isolated bacteria have shown a positive result for ACC deaminase production, phosphate solubilization, and catalase enzyme secretion. The authors evaluated the performance of both the bacteria and their consortium under high temperature, drought, and salinity and observed positive responses, and thus has the potential to be explored for improved agricultural production of common beans.

Excess generation of reactive oxygen species (ROS) is a known consequence of most environmental stress signals. Vu et al. showed that the (ROS)-mediated plasmodesmal regulation requires a network of an Arabidopsis receptor-like kinase, calmodulin-like proteins, and callose synthases. Authors showed that the Arabidopsis NOVEL CYS-RICH RECEPTOR KINASE (NCRK), a plasmodesmata-localized protein, is required for plasmodesmal callose deposition in response to ROS stress. Further, NCRK interacted with calmodulin-like protein 41 (CML41) and GLUCAN SYNTHASE-LIKE 4 (GSL4) in response to ROS stress. The results are significant since plasmodesmata is considered critical for symplasmic communication, and in coordinating plant growth, development, and environmental stress responses. The study holds importance and the proposed model can be explored further.

Understanding the genetic makeup of traits associated with drought stress tolerance in bread wheat at seedling and reproductive phases is essential for developing drought-tolerant varieties. Reddy et al. selected 192 diverse wheat genotypes to evaluate their performance at the seedling stage in a hydroponics system for assessing physiological parameters under both drought and optimum conditions. They further carried out a genome-wide association study (GWAS) using the phenotypic data recorded during the hydroponics experiment as well as data available from previously conducted multi-location field trials. The GWAS identified 94 significant marker-trait associations (MTAs) or SNPs associated with traits recorded at the seedling stage and 451 for traits recorded at the reproductive stage. Identified SNPs represented several novel, significant, and promising MTAs for different traits. Functional annotation and in silico studies revealed important putative candidate genes underlying the identified stable genomic regions such as protein kinases, O-methyltransferases, GroES-like superfamily proteins, NAD-dependent dehydratases, etc. These findings are important and may be explored for improving yield potential and stability under drought stress conditions.

Future climatic CO2 levels are shown to affect plant growth and its interactions with other stresses. Shabbaj et al. studied the alleviating effect of increasing atmospheric CO2 levels on heavy metal toxicity. Heavy metal contamination, especially with In_2_O_3_-NPs has become a serious concern for the ecosystem and is a challenge for plant growth and productivity. The authors showed how soil contamination with In_2_O_3_-NPs affects growth of C3 (wheat) and C4 (Sorghum) plants treated with future climate CO2 and In_2_O_3_-NPs, and how CO_2_ enrichment differentially upregulated sugar, proline, and polyamine metabolism in young and old leaves of wheat and sorghum to mitigate In_2_O_3_-NPs toxicity. The induced levels of these metabolites were reported to be involved in the osmo- and redox regulation to reduce NP-induced oxidative damage. The study has suggested that eCO_2_ can be useful in modulating carbon and nitrogen metabolism in both C3 and C4 plants to improve their toxicity tolerance against nanoparticle toxicity.

Overall, the ‘Research Topic’ has exemplified the need for continued research into plant environmental stress tolerance in crop plants. Current updates and recent advances in the physiological, molecular, and genetic perspectives of plant responses to single and combined abiotic stresses may offer insights underlying these responses and how this pool of knowledge can be explored to develop plants for future climate. More research inputs are required to understand the combinatorial stress, as a new stressor through molecular-physiological, phenomics and other omics studies. Detection of the stressor and perception of the sensing genetic cues will be crucial for improving plant stress tolerance and maintaining yield potential. Presently large data has been generated using omics studies, however several genomic and metabolomic signatures from this data need to be validated for their functional role in stress recovery and tolerance responses to combined and/or sequential stresses. There is a need to employ diverse stress tolerance traits for their contribution to higher productivity in challenged environments to aid future food security issues. Alternatively, the role of plant microbiome in alleviating abiotic combinatorial stress conditions needs to be investigated through extensive field based trials in crop plants. The advances in discovery of candidate genes, GWAS-defined QTLs and targeted genome editing, could be used to develop climate resilient, future-ready stress tolerant varieties. Experimental environmental models that mimic combined stress under natural field conditions will have to be developed for testing the genetically engineered lines. There has been extensive research in plant abiotic stress biology especially to salinity, drought, heat, cold and heavy metal stress, however there is always co-occurrence of different stresses under realistic field conditions, and hence a paradigm shift is warranted for the development of tolerance to sequential or combined abiotic/biotic stresses, and testing the tolerant lines under natural stress conditions.

## Author contributions

SP: Writing – review & editing. AS: Writing – review & editing. VK: Writing – original draft, Writing – review & editing. OS: Writing – review & editing.
